# An Unusual Progression of Membranous Nephropathy

**DOI:** 10.7759/cureus.30651

**Published:** 2022-10-25

**Authors:** Arundhati Sharma, Anubhuti Sharma, Kartik Upreti, Swetha Movva, D Ragasri Meghana, Chinmay Khotele, Sangamesh N Malipatil, Devarsh N Shah, Vishal Venugopal

**Affiliations:** 1 Medical School, Institute of Medical Sciences, Banaras Hindu University, Varanasi, IND; 2 Medical School, Vardhman Mahavir Medical College and Safdarjung Hospital, New Delhi, IND; 3 Pediatrics, Narayana Medical College, Nellore, IND; 4 Medical School, Kakatiya Medical College, Warangal, IND; 5 Internal Medicine, Indira Gandhi Government Medical College, Nagpur, IND; 6 Medical School, Mahadevappa Rampure Medical College, Kalaburagi, IND; 7 Medicine and Surgery, Medical College Baroda, Vadodara, IND; 8 Internal Medicine, Bhaarath Medical College and Hospital, Chennai, IND

**Keywords:** rituximab, ace inhibitor, membranous nephropathy, nephrotic syndrome, glomerulonephritis

## Abstract

Membranous glomerulopathy is one of the commonest causes of nephrotic syndrome and chronic renal insufficiency in adults. There has been documented evidence of a poorer prognosis with factors such as male gender, advanced age, increased blood pressure and persistent loss of proteins in the urine, but the overall prognosis of this condition is excellent. Herein, we present the case of a 20-year-old female patient who was diagnosed with primary membranous nephropathy. Normally, cases of primary membranous nephropathy have good outcomes with conservative management and immunosuppressants but our case had a worsening course and a delayed response even with immunosuppressive treatment. This case has been recorded due to its unusual presentation, unnatural course, and outcomes contrary to what is seen in routine clinical practice.

## Introduction

The most likely cause of nephrotic syndrome in adults (40-60 years of age) is membranous nephropathy (MN). Nephrotic syndrome persists in almost half of the patients diagnosed with MN and around one-third of patients with MN progress to end-stage renal disease [1,2]. MN is usually accompanied by low-grade blood loss in urine and relatively well-preserved kidney function, manifesting as typical nephrotic syndrome with heavy proteinuria as the major investigative finding. On routine urine analysis, more than 75% of these patients have mild to moderate grade proteinuria (less than 500mg/day) or nephrotic-range proteinuria (more than 3-3.5 g/day), with only a few developing severe impairment, losing about 15-20 g of protein per day [1].

Most cases are primary or have no known cause, and the phospholipase A2 receptor of type-M is a target antigen in 70% of people [3]. Secondary MN can be due to the use of specific drugs such as penicillin, gold, or less frequently, ACE inhibitors like captopril or even nonsteroidal anti-inflammatory drugs. It can also be linked to infections like hepatitis B and C, neoplasms of the breast, lung, or gastrointestinal system, or sometimes both. MN can occasionally develop in people with existing autoimmune diseases such as systemic lupus erythematosus (class five lupus nephritis) [1,2]. Normal levels of serum complements are typical in patients with idiopathic MN. The glomeruli often seen are normocellular under light microscopy with thickening of the glomerular basement membrane (GBM). Additional "spike-like" protrusions, which are made of a material resembling basement membrane, can be seen on the epithelial side of GBM when silver methenamine is used. IgG and C3 deposits are present as a "granular" pattern along the capillary wall under immunofluorescence microscopy and are found in sub-epithelial areas under electron microscopy [2].

The management of MN includes medical therapy (diuretics, ACE inhibitors, lipid control drugs, and immunosuppressants) in combination with control of blood pressure, restriction of salt and proteins in the diet, and even the use of prophylactic anticoagulants in some cases. Severe cases which have progressed to end-stage renal disease require a renal transplant but the recurrence rate is very high [4].

## Case presentation

Clinical course

A 20-year-old female presented to the outpatient department with complaints of swelling in both the lower extremities and face. Her history revealed that facial swelling began one month ago. There has also been an associated history of weight gain since a month, as well as frothy urine for the past 2 weeks. The patient did not have any history of visible blood in the urine or a complaint of reduced urine output. The patient also did not have any history of rashes, joint pain, oral ulceration, Raynaud's phenomenon or any other complaints suggestive of a connective tissue disorder. There had been no previous history of a similar presentation, no personal or family history of thyroid, renal, connective tissue or autoimmune disorders, and no associated co-morbidities such as systemic hypertension or diabetes mellitus. In addition, the patient did not report any recent sore throats or fever, or any medication history.

On examination, the patient showed facial puffiness along with bilateral pedal edema, and the remaining systemic examination was otherwise unremarkable. The patient was subjected to subsequent investigations. Laboratory results (Table 1) showed elevated 24-hour urinary protein on urine analysis. Blood reports were sent for lipid and thyroid profiles which came out to be relatively abnormal. The complement levels were found to be within the normal range. Transthoracic echocardiography and renal ultrasonography reports were also normal. Nephrotic syndrome was kept as the differential since the patient presented with bilateral lower limb edema, frothy urine and elevated blood pressure. Polycystic kidney disease and renal artery stenosis were ruled out with a normal renal USG. The patient is not diabetic, which ruled out diabetic nephropathy. The patient was prescribed diuretics and statins and was discharged with instructions to return in two weeks. 

**Table 1:** Laboratory investigation report during the first visit aPTT: activated partial thromboplastin clotting time; WBC: white blood cell; PCV: packed cell volume; RBC: red blood cell; MCV: mean corpuscular volume; MCH: mean corpuscular hemoglobin; MCHC: mean corpuscular hemoglobin concentration; ESR: erythrocyte sedimentation rate; LDL: low-density lipoprotein; HDL: high-density lipoprotein; TSH: thyroid stimulating hormone

Parameter	Patient Values	Normal Value
COMPLEMENT LEVEL		
C3 Level	190 mg/dL	90-180 mg/dL
C4 Level	40 mg/dL	10-40 mg/dL
RENAL FUNCTION TESTS		
24 hr Urine Protein	3216 mg/24hour	< 140 mg/24hour
S. Albumin	3.3 g/dL	3.5-5.5 g/dL
S. Urea	11.9 mg/dL	15-45 mg/dL
S. Creatinine	0.59 mg/dL	0.80-1.30 mg/dL
Sodium	138 mmol/L	136-145 mmol/L
Potassium	4.7 mmol/L	3.5-5.1 mmol/L
Chloride	106 mmol/L	98-107 mmol/L
Bicarbonate	25 mmol/L	21-30 mmol/L
COAGULATION PROFILE		
Prothrombin Time (PT)	11 seconds	10-15 seconds
aPTT	25.5 seconds	21-38 seconds
COMPLETE BLOOD COUNT		
Total WBC Count	7100 cells/mm3	4000-11000 cells/mm3
Neutrophils	54 %	40-70 %
Lymphocytes	33 %	20-40 %
Eosinophils	10 %	0-8 %
Monocytes	2 %	1-10 %
Basophils	0 %	0-1 %
Haemoglobin	9.8 g/dL	11-15 g/dL (adult female)
PCV	34.5 %	35-48 %
Total RBC Count	5.2 Million/mm3	3.8-5.8 million/mm3
MCV	67 fL	80-100 fL
MCH	19 pg	27-31 pg
MCHC	28 %	31-35 %
Platelet Count	1.9 Lakhs/mm3	1.5-4.5 lakhs/mm3
ESR (1hr)	82 mm	0-22 mm
LIPID PROFILE		
Total Cholesterol	304.5 mg/dL	<170 mg/dL
HDL Cholesterol	45.3 mg/dL	40-60 mg/dL
Triglycerides	317.5 mg/dL	<150 mg/dL
LDL Cholesterol	195.7 mg/dL	<129 mg/dL
THYROID PROFILE		
S. TSH	7.15 micro IU/mL	0.35-5 micro IU/mL
Total T4	6.37 mcg/dL	4.6-12 mcg/dL
Total T3	127.5 mg/dL	80-200 mg/dL

**Figure 1:**
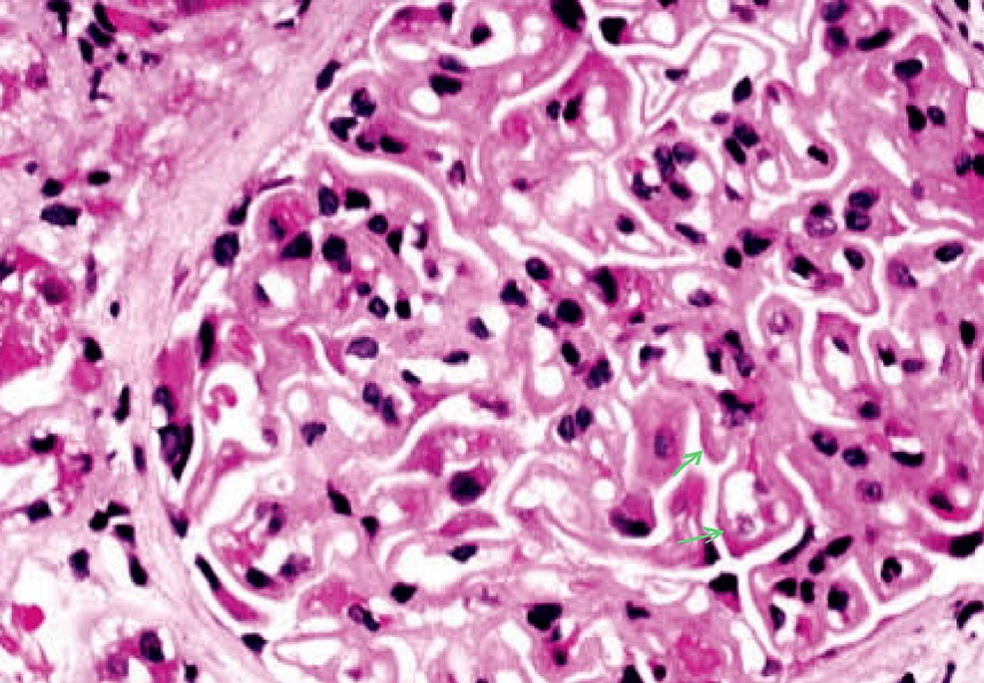
Light microscopy findings (green arrow shows rigid and thickened capillary loops)

At the follow-up visit after two weeks, patient findings had barely subsided, and therefore further investigations were ordered to understand the underlying aetiology of nephrotic syndrome. Blood investigations on the second visit (Table 2) showed improvements in lipid profiles but worsening proteinuria and renal function as compared to the baseline. Antibody tests were done for anti-nuclear antibodies (ANA), which turned out to be negative, and also for serum anti-PLA2R auto-antibodies which came out to be positive. A core needle renal biopsy was ordered during the second visit and the light microscopy findings (Figure 1) showed the presence of spikes on the glomerular basement membrane and an absence of fibrosis, which pointed towards membranous glomerulonephropathy among nephrotic syndromes. The absence of any inflammatory infiltrate in the interstitium was helpful in ruling out a nephritic syndrome etiology. Immunofluorescence studies showed the presence of IgG and C3 on the capillary loops with sub-epithelial deposition of immune complexes. The patient was then started on rituximab, which is an immunosuppressant, and eventually, the patient's condition improved after an aggressive course.

**Table 2:** Laboratory investigation report during the second visit. aPTT: activated partial thromboplastin clotting time; WBC: white blood cell; PCV: packed cell volume; RBC: red blood cell; MCV: mean corpuscular volume; MCH: mean corpuscular hemoglobin; MCHC: mean corpuscular hemoglobin concentration; ESR: erythrocyte sedimentation rate; LDL: low-density lipoprotein; HDL: high-density lipoprotein

Parameter	Patient Values	Normal Value
COMPLEMENT LEVEL		
C3 Level	155 mg/dL	90-180 mg/dL
C4 Level	30 mg/dL	10-40 mg/dL
RENAL FUNCTION TESTS		
24 hr Urine Protein	5719 mg/24hour	< 140 mg/24hour
S. Albumin	2.7 g/dL	3.5-5.5 g/dL
S. Urea	15.9 mg/dL	15-45 mg/dL
S. Creatinine	0.8 mg/dL	0.80-1.30 mg/dL
Sodium	142 mmol/L	136-145 mmol/L
Potassium	4.5 mmol/L	3.5-5.1 mmol/L
Chloride	106 mmol/L	98-107 mmol/L
Bicarbonate	26 mmol/L	21-30 mmol/L
COAGULATION PROFILE		
Prothrombin Time (PT)	13.4 seconds	10-15 seconds
aPTT	31.6 seconds	21-38 seconds
COMPLETE BLOOD COUNT		
Total WBC Count	7100 cells/mm3	4000-11000 cells/mm3
Neutrophils	58 %	40-70 %
Lymphocytes	34 %	20-40 %
Eosinophils	6 %	0-8 %
Monocytes	2 %	1-10 %
Basophils	0 %	0-1 %
Haemoglobin	8.9 g/dL	11-15 g/dL (adult female)
PCV	29 %	35-48 %
Total RBC Count	4.3 Million/mm3	3.8-5.8 million/mm3
MCV	67 f/L	80-100 f/L
MCH	21 pg	27-31 pg
MCHC	31 %	31-35 %
Platelet Count	3.1 Lakhs/mm3	1.5-4.5 lakhs/mm3
ESR (1hr)	32 mm	0-22 mm
LIPID PROFILE		
Total Cholesterol	260 mg/dL	<170 mg/dL
LDL cholesterol	180 mg/dL	<129 mg/dL
HDL cholesterol	44 mg/dL	40-60 mg/dL
Triglycerides	302 mg/dL	<150 mg/dL

Biopsy results

**Figure 2:**
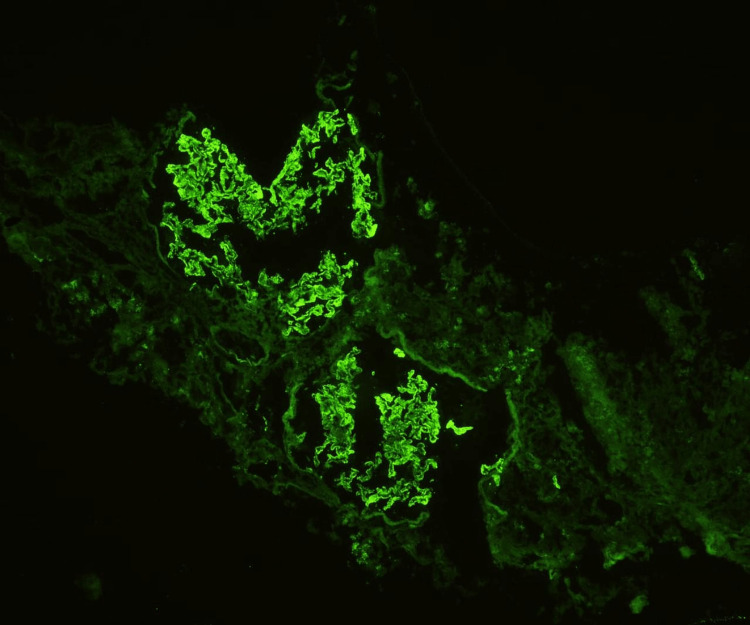
Granular staining pattern on immunofluorescence microscopy.

A piece of tissue measuring 0.4×0.6cm preserved in formalin was sent for light microscopy, which showed 17 glomeruli and none were globally sclerotic. Cellularity was normal with a patent capillary lumen. There was spike formation in the glomerular basement membrane and thickening of capillary walls, but no segmental sclerosis, endocapillary hyper-cellularity, or crescent formation was observed (Figure 1). There were no signs of interstitial fibrosis or tubular atrophy, and there were no inflammatory cells in the interstitial space. A piece of tissue measuring 0.3×0.6cm in Michel's Medium was sent for immunofluorescence. Eight glomeruli were considered for evaluation, and sections were stained for antibodies such as IgG, IgM, IgA, complement C3 and C1q, and even the light chains kappa and lambda. The result was positive for IgG (+3) and C3 (+2) and showed granular positivity on the capillary loops (Figure 2), but negative for the rest of the components.

Management

Initial management included oral torsemide 5 mg once daily for three days and rosuvastatin 10 mg once daily for one week. During the follow-up visit, the patient showed mild improvement in lipid profile and swelling but worsening in proteinuria and renal function tests (RFTs) as compared to her baseline values from the first visit. The patient was then prescribed telmisartan 40 mg once daily and rituximab 375 mg once a week for a period of four weeks. The patient was followed up with regular phone calls but there was not much improvement even after two weeks. The dosage of telmisartan was increased to 80 mg once daily without any changes in rituximab dosage, and during the third visit, after a period of four weeks, the patient showed significant clinical improvement (reduction in facial puffiness, lower limb edema and frothy urine) as well as improvement in lab reports, with proteinuria returning to the normal value (less than 150 mg/day) and RFTs returning to their baseline value.

## Discussion

Membranous nephropathy (MN) is found in up to one-third of the biopsied cases of nephrotic syndrome, making it one of the most common causes of nephrotic syndrome, especially in nondiabetic adults [1]. Clinical presentation includes peripheral edema, frothy urine, hypertension, and even features suggesting thromboembolic states in severe cases. Laboratory investigations may show hypoalbuminemia, dyslipidemia, massive proteinuria (>3.5g/day) and acute kidney injury with elevated serum creatinine levels.

Three possible mechanisms have been suggested in the pathogenesis of MN. The first one is the direct glomerular deposition of the immune complexes that have already formed in the blood circulation. Second, the circulating antibodies react with an antigen intrinsic to the glomeruli, which is not continuously distributed along the laminar membrane adjacent to the podocytes, resulting in the formation of immune complexes directly in the glomeruli. The third possibility is that there is a reaction of circulating antibodies with an antigen not intrinsic to the glomerulus but deposited there due to some kind of affinity for the glomerular basement membrane [1]. These things upset the structure of the podocytes, hurt the integrity of the slit diaphragm, and cause the negatively charged membrane barrier to break down, which leads to a lot of protein in the urine [5]. 

Renal biopsy is the gold standard investigation for diagnosing the cause of nephrotic syndrome. The characteristic lesion on light microscopy, which was seen in our patient, is the diffusely thickened GBM and capillary walls throughout all the glomeruli without any significant hypercellularity. A diffuse granular staining pattern of IgG and C3 is seen along the GBM on immunofluorescence microscopy. Electron microscopy shows electron-dense deposits on the subepithelial outer segment of the GBM, which is considered the hallmark lesion. It also shows the effacement of podocyte foot processes, and GBM expansion due to the accumulation of new matrix extracellularly in between these deposits (which appear as "spikes" on visualising with special stains) [2]. Some studies indicate that, for the progression of the disease, the degree of atrophy in the tubules or the degree of fibrosis in the interstitium is more predictive than the stage of glomerular disease. Some studies also suggest that an elevated titer of antibodies such as anti-PLA2R, as was in our case, is associated with a lower response to immunosuppressive therapy and a longer time to normal state [6]. 

There is a need for effective and prompt therapy with favourable outcomes for patients diagnosed with MN, and a more personalised approach should be sought for the management of these patients. Treatment for patients diagnosed with MN should be directed towards improving the elevated blood pressure, reducing the generalised or pedal edema, and limiting the risks of thromboembolism and adverse cardiovascular events. Complete proteinuria remission has been shown to significantly improve long-term survival in MN patients, preventing the progression to renal failure. Good long-term results are also seen with partial remission, which stops the kidney function from getting worse [7]. ACE inhibitors, or angiotensin II receptor blockers (ARB), are preferred antihypertensives over the other classes of blood pressure-lowering drugs due to their proven renoprotective effects and substantial potential to reduce proteinuria. In our case, we saw a prominent improvement in outcomes after increasing the dose of an ACE inhibitor. Patients with MN and nephrotic syndrome, especially those with albumin levels of 2.2 g/dl, are more likely to have thromboembolic events. This suggests the need for prophylactic anticoagulation therapy [4,8].

Some patients like ours are not responsive to conservative treatment, and hence alternative approaches are sometimes required to prevent worsening outcomes. KDIGO guidelines (Kidney Disease Improving Global Outcomes) suggest that immunotherapy should be considered when patients have persistent proteinuria, declining kidney function, or severe symptoms [8]. Alternating alkylating agents plus intravenous pulse or oral corticosteroids have been shown to be effective in treating MN (Ponticelli regimen). A six-month cycle could potentially aid in returning the patient to complete normal [9]. Some recent studies have even demonstrated the effectiveness of rituximab for the treatment of MN. It is a monoclonal IgG1 antibody that has an effect on B-cell depletion by binding and inhibiting CD20. It is often used to treat lymphomas, ANCA-associated vasculitis, rheumatoid arthritis, and other autoimmune diseases like membranous nephropathy, which was the case in our report [10,11]. In our case, we preferred the initial immunosuppressive treatment with rituximab over corticosteroids and the cyclosporine regimen due to previous positive clinical experiences. 

## Conclusions

Our patient presented with signs and symptoms of acute nephrotic syndrome. She was started on conservative management with diuretics and statins but did not show any improvements during the follow-up visit. The patient was diagnosed with primary membranous nephropathy at the follow-up visit based on biopsy and antibody test results. For future such cases, we would suggest an earlier biopsy at the initial visit itself along with the initiation of specific medical therapy pertaining to the diagnosis. Appropriate treatment should be aimed at the reduction of proteinuria, which is the single most important factor determining renal functioning. The focus should be gradually shifted towards newer and relatively safer treatment modalities like immunomodulators and monoclonal antibodies.
